# Identification and characterization of a novel fumarase gene by metagenome expression cloning from marine microorganisms

**DOI:** 10.1186/1475-2859-9-91

**Published:** 2010-11-23

**Authors:** Chengjian Jiang, Lan-Lan Wu, Gao-Chao Zhao, Pei-Hong Shen, Ke Jin, Zhen-Yu Hao, Shuang-Xi Li, Ge-Fei Ma, Feng-Feng Luo, Guo-Qing Hu, Wen-Long Kang, Xing-Mei Qin, You-Li Bi, Xian-Lai Tang, Bo Wu

**Affiliations:** 1 Guangxi Key Laboratory of Subtropical Bioresources Conservation and Utilization, The Key Laboratory of Ministry of Education for Microbial and Plant Genetic Engineering, College of Life Science and Technology, Guangxi University, 100 Daxue East Road, Nanning, Guangxi, 530004, People's Republic of China; 2 College of Chemistry and Ecology Engineering, Guangxi University for Nationalities, 188 Daxue East Road, Nanning, Guangxi, 530006, People's Republic of China

## Abstract

**Background:**

Fumarase catalyzes the reversible hydration of fumarate to L-malate and is a key enzyme in the tricarboxylic acid (TCA) cycle and in amino acid metabolism. Fumarase is also used for the industrial production of L-malate from the substrate fumarate. Thermostable and high-activity fumarases from organisms that inhabit extreme environments may have great potential in industry, biotechnology, and basic research. The marine environment is highly complex and considered one of the main reservoirs of microbial diversity on the planet. However, most of the microorganisms are inaccessible in nature and are not easily cultivated in the laboratory. Metagenomic approaches provide a powerful tool to isolate and identify enzymes with novel biocatalytic activities for various biotechnological applications.

**Results:**

A plasmid metagenomic library was constructed from uncultivated marine microorganisms within marine water samples. Through sequence-based screening of the DNA library, a gene encoding a novel fumarase (named FumF) was isolated. Amino acid sequence analysis revealed that the FumF protein shared the greatest homology with Class II fumarate hydratases from *Bacteroides *sp. 2_1_33B and *Parabacteroides distasonis *ATCC 8503 (26% identical and 43% similar). The putative fumarase gene was subcloned into pETBlue-2 vector and expressed in *E. coli *BL21(DE3)pLysS. The recombinant protein was purified to homogeneity. Functional characterization by high performance liquid chromatography confirmed that the recombinant FumF protein catalyzed the hydration of fumarate to form L-malate. The maximum activity for FumF protein occurred at pH 8.5 and 55°C in 5 mM Mg^2+^. The enzyme showed higher affinity and catalytic efficiency under optimal reaction conditions: *K*_m_= 0.48 mM, *V*_max _= 827 μM/min/mg, and *k*_cat_/*K*_m _= 1900 mM/s.

**Conclusions:**

We isolated a novel fumarase gene, *fumF*, from a sequence-based screen of a plasmid metagenomic library from uncultivated marine microorganisms. The properties of FumF protein may be ideal for the industrial production of L-malate under higher temperature conditions. The identification of FumF underscores the potential of marine metagenome screening for novel biomolecules.

## Background

Fumarase, or fumarate hydratase, (EC 4.2.1.2) is a critical enzyme of the tricarboxylic acid (TCA) cycle, where it catalyzes the reversible hydration of fumarate to L-malate [1]. Currently, fumarases from some mesophilic microorganisms, such as *Lactobacillus brevis *and *Corynebacterium glutamicum*, have been exploited for the industrial production of L-malate using fumarate as a substrate [2]. In the L-malate production process, fumarases are often inactivated at higher temperatures (40-60°C) and by metal ions, requiring constant replenishment of the biocatalyst [3]. Mining for thermostable and high-activity fumarases from extreme environments could improve industrial L-malate production.

There are two distinct classes of fumarases, classified according to subunit composition, thermal stability, and metal requirements [4]. The Class I fumarases, encoded by the *fumA *and *fumB *genes, are thermolabile homodimers containing an Fe-S cluster that may catalyze the reduction of L-malate by providing fumarate as the anaerobic electron acceptor [5]. The FumA protein is stable, whereas FumB protein is unstable under aerobic conditions and functions only under anaerobic conditions [6,7]. The Class II fumarases, encoded by the *fumC *gene, are thermostable homotetramers with no requirement for cofactors and catalyze the interconversion of fumarate to L-malate [2]. FumC proteins are widely distributed in nature, from prokaryotes like *Bacillus subtilis*, *Pseudomonas aeruginosa*, *Sulfolobus solfataricus*, and *Saccharomyces cerevisiae*, to mammals [3,8].

A large number of studies have focused on fumarases from specific species of plants, mammals, and microorganisms, but very few have studied this enzyme in uncultivated marine microorganisms. It is widely accepted that the marine environment possesses unique microbial diversity, and so are a vast resource for mining novel genes and biocatalysts [9]. Ninety-nine percent of marine microorganisms are not readily cultivated using currently available laboratory techniques and so are not accessible to the biotechnology industry or to basic researchers [10]. The collective genomes of all microorganisms present in a given habitat, the so-called metagenome [11,12], has been screened for biocatalysts and other biomolecules for new biotechnological applications or simply to understand the microbial ecology and physiology of the marine environment. The most important advantage of the metagenomic library is that it contains genomes from many species of microorganisms; thus, it provides a more comprehensive collection of global microbiological information [13]. Currently, various industrial enzymes, including esterases or lipases, proteases, amylases, nitrilases, and lyases, have been identified through the metagenomic approach [14,15].

In this study, a plasmid metagenomic library was constructed from marine water samples. Through sequence-based screening of the DNA library, a gene encoding a novel fumarase (named FumF) was isolated. The deduced amino acid sequence exhibited moderate similarity to a family of Class II fumarase proteins. Biochemical analysis of the expressed recombinant protein revealed that FumF catalyzed the hydration of fumaric acid to form L-malate. To our knowledge, this is the first report on a fumarase isolated from a marine metagenome.

## Results and Discussion

### Construction of marine microbial metagenomic library

Metagenomic DNA was directly extracted from marine water samples. The constructed library should contain genes from all the microorganisms in the sample, including species that have not been classified or that cannot be cultivated. Approximately 50-80 μg of crude DNA extract was obtained from 100 L of sea water. Most of the DNA molecules were about 23 kb as revealed by a strong band on agarose gels (data not shown). A similar DNA yield with approximately the same dominant band (≈23 kb) was reported following marine water DNA isolation in the previous study [16]. The crude extract was separated on a low-melting agarose gel to further purify the DNA. The DNA collected from the agarose gel was partially digested with *Eco*RI and ligated with the *Eco*RI-cleaved pGEM-3Zf(+) vector. The final plasmid metagenomic library was constructed from 10 μg of purified sea water DNA and contained approximately 50,000 clones. Restriction analysis of randomly chosen recombinant plasmids revealed a high level of diversity of foreign DNA fragments in pGEM-3Zf(+). When *Eco*RI-digested plasmids from 11 randomly picked clones were analyzed by agarose gel electrophoresis, the sizes of the inserts ranged from 1-15 kb, with an average of about 4.0 kb (see Additional file 1). These results indicated that the marine library covered ≈200 Mb and that screening the metagenome DNA library could yield novel functional genes [17,18].

### Bioinformatics analysis and cloning of a fumarase gene

The metagenome plasmid library was grown on microplates. Bacterial cultures were amplified, pelleted, and lysed. Randomly chosen recombinant plasmids were sequenced and analyzed. The DNA sequence analysis and database search revealed that three out of the 15 open reading frames (ORFs) of predicted genes exhibited over 80% similarity to known genes, while the other 12 recombinant plasmids did not match any known sequence in the database (detailed data not shown). This result confirmed that the marine metagenomic library contained DNA molecules from uncharacterized genomes, and demonstrated that the marine metagenome of naturally occurring bacteria contained an immense pool of genes, most of which are not represented in pure or enriched cultures established under selective conditions [19].

Using a sequence-based strategy, we screened the metagenomic library and identified an interesting recombinant clone, pGXAM3566, that may encode a fumarase enzyme. The insert DNA had a length of 2011 bp, and showed no good matches at the DNA level with known genes in the database. However, pGXAM3566 showed moderate homology to some fumarate hydratases at the amino acid level. Based on the database comparison, we considered the cloned gene pGXAM3566 novel and named it *fumF*. The gene had a long ORF of 1371 bp (G + C content of 57.8%) that encoded a 457 amino acid protein. The predicted Mr was approximately 49.2 kDa with an isoelectric point of 4.8, similar to the 48-50 kDa Mr of most FumC proteins [7]. When the deduced amino acid sequence of FumF was searched against the NCBI and Expasy databases, FumF protein was found to share partial homology with the Class II fumarate hydratases from *Bacteroides *sp. 2_1_33B and *Parabacteroides distasonis *ATCC 8503 (26% identical and 43% similar). Other Class II fumarate hydratases with partial homology were fumarate hydratases from *Parabacteroides *sp. D13 (GenBank accession no. ZP_05544942; 25% identical and 42% similar), *Bacteroides *sp. 3_1_19 (GenBank accession no. ZP_06985098; 25% identical and 42% similar), and *Bacteroides *sp. 2_1_7 (GenBank accession no. ZP_05287561; 25% identical and 42% similar).

**Figure 1:**
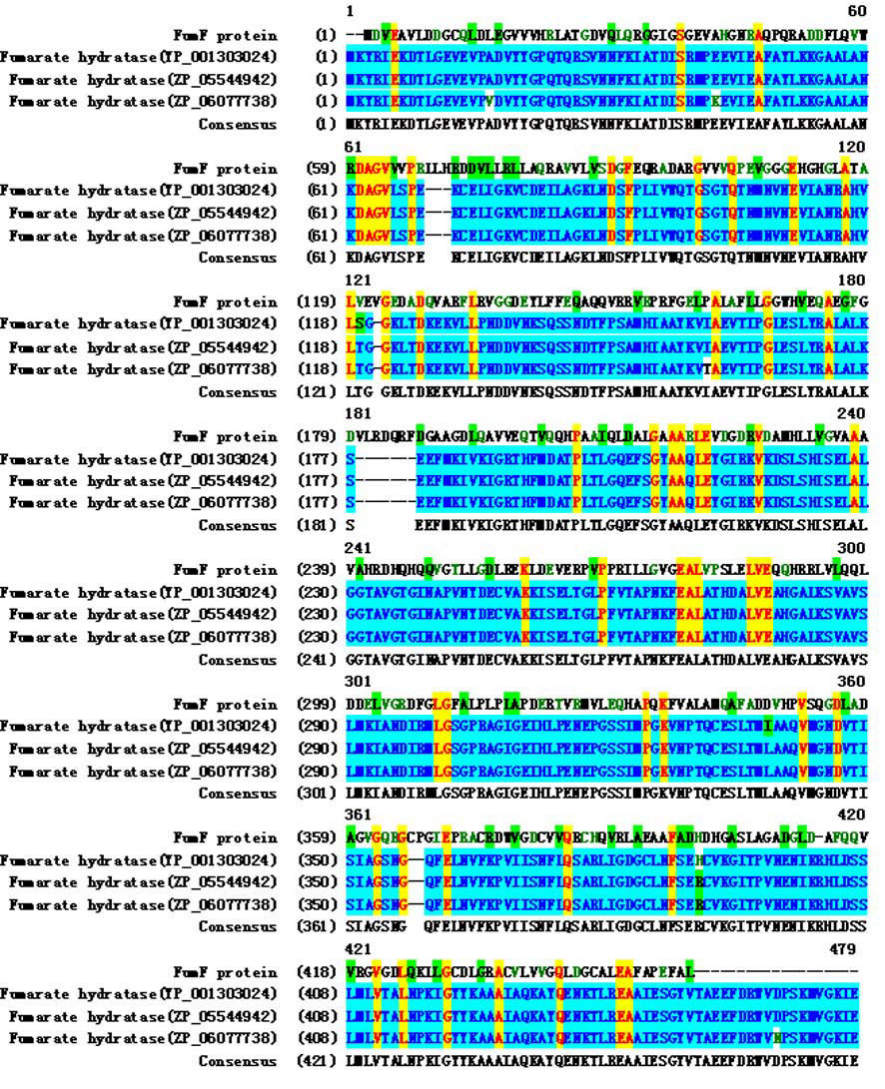
**Sequence alignment of FumF protein with other fumarases**. Fumarases are identified by their GenBank accession numbers. Sequence similarity searches were performed with the BLAST 2.0 program. Amino acid sequence alignment of the target putative protein with homologous proteins was performed with the Align × program, a component of the Vector NTI suite (Informax, North Bethesda, MD, USA), using the blosum62mt2 scoring matrix.

Multiple alignments with the most homologous fumarases proteins (NCBI database) are presented in Figure 1. The FumF protein showed greater homology with bacterial fumarases than with eukaryotic homologues. Amino acid sequence comparison revealed that the deduced FumF peptide shared the conserved active site residues of other Class II fumarases. In contrast, an amino acid N-terminal extension was present only in the bacteria fumarases (Figure 1). Based on amino acid sequence homology, FumF may be a new member of the Class II fumarate hydratases [7].

**Figure 2:**
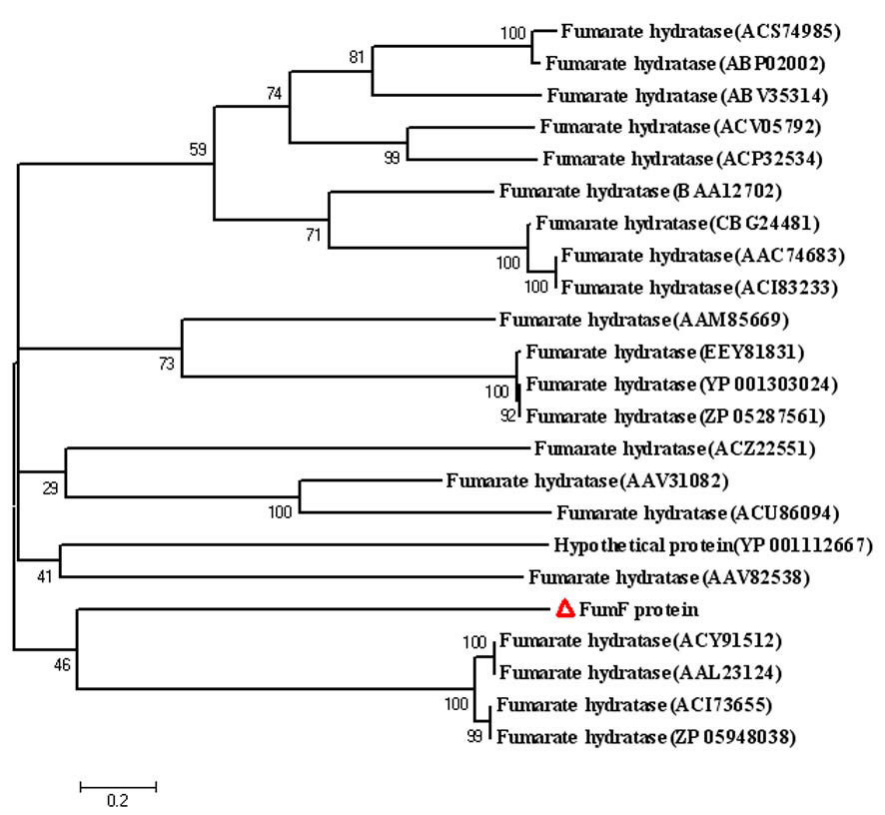
**Phylogenetic relationship of FumF protein with other fumarases**. Sequence alignment was performed using ClustalW version 1.81, and the phylogenetic tree was constructed by the neighbor-joining method using MEGA version 4.0 [30]. Boot-strapping values were used to estimate the reliability of phylogenetic reconstructions (1,000 replicates). The numbers associated with the branches refer to bootstrap values (confidence limits) representing the substitution frequencies per amino acid residue. Fumarases are identified by their GenBank accession numbers.

A phylogenetic tree based on the neighbor-joining method place FumF protein in a distinct clade from the Class II fumarases found in other microorganisms (Figure 2). Such a placement suggests a relatively high level of divergence for the Class II fumarases. The FumF protein was also not grouped with eukaryotic homologues, reflecting considerable dissimilarities between the eukaryotes and prokaryotes furmarases. Sequence comparison between FumF protein and others FumCs revealed many differences clustered at the 5' end of the coding sequence.

### Expression and purification of recombinant FumF protein

**Figure 3:**
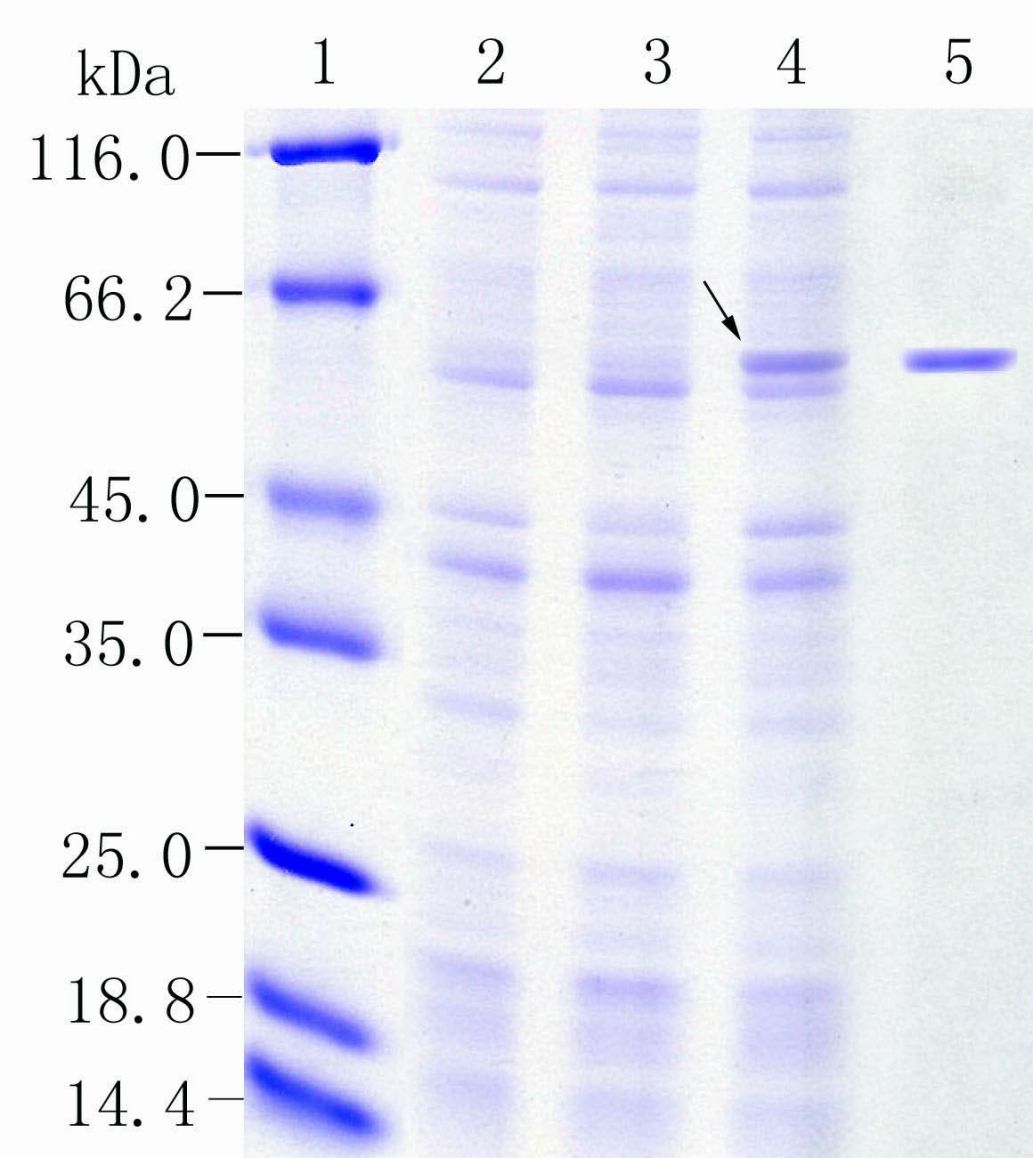
**SDS-PAGE of recombinant FumF protein**. Proteins were separated by 12% (w/v) SDS-PAGE and then stained with Coomassie brilliant blue G-250. Lane 1, molecular weight standards; Lane 2, total protein of *E. coli *BL21(DE3)pLysS harboring empty pETBlue-2 (control); Lane 3, total protein of *E. coli *BL21(DE3)pLysS harboring the recombinant *fumF *in pETBlue-2 without induction by IPTG; Lane 4, total protein of *E. coli *BL21(DE3)pLysS harboring the recombinant *fumF *in pETBlue-2 induced by addition of 0.5 mM IPTG; Lane 5, sample purified by the Ni-NTA column method. The recombinant FumF protein is indicated by the black arrow.

To investigate the biochemical properties of FumF, the gene was subcloned in frame with a six-histidine tag sequence into the expression vector pETBlue-2 and expressed in *E. coli *BL21(DE3)pLysS. The initial analysis of the crude cell lysate revealed that the bacteria containing recombinant plasmid pETBlue-2-*fumF *produced a substantial amount of the expected recombinant protein, while FumF was not detectable in bacteria containing the parent vector pETBlue-2. Protein extracts were subjected to SDS-PAGE. Increased expression of a ≈ 55 kDa protein was observed in cell extracts of recombinant FumF. The molecular weight of the protein was similar to recombinant FumF, indicating that FumF was probably expressed intracellularly without extensive modifications. The recombinant FumF protein was then purified by Ni-NTA Magnetic Agarose Chromatography (Figure 3). The separated proteins produced a single band on SDS-PAGE gels, and the molecular weight concurred with that deduced from the amino acid sequence of the recombinant protein.

### Functional characterization and physicochemical characterization of recombinant FumF protein

We confirmed that the purified recombinant FumF protein exhibited fumarase activity by HPLC analysis of the reaction product (see Additional file 2). The retention time of the enzymatic product was 7.369 min, matching that of an L-malate standard (7.4 min), and different with the substrate fumarate peak.

The functional characterization of FumF may provide new insights into the relationship between sequence, structure, and activity of Class II fumarases [20]. Furthermore, functional characterization could reveal novel uses for FumF. Elucidating the catalyzing mechanism through studies of the 3 D structures of FumF protein with X-ray diffraction methods is also an interesting direction for future research.

**Figure 4:**
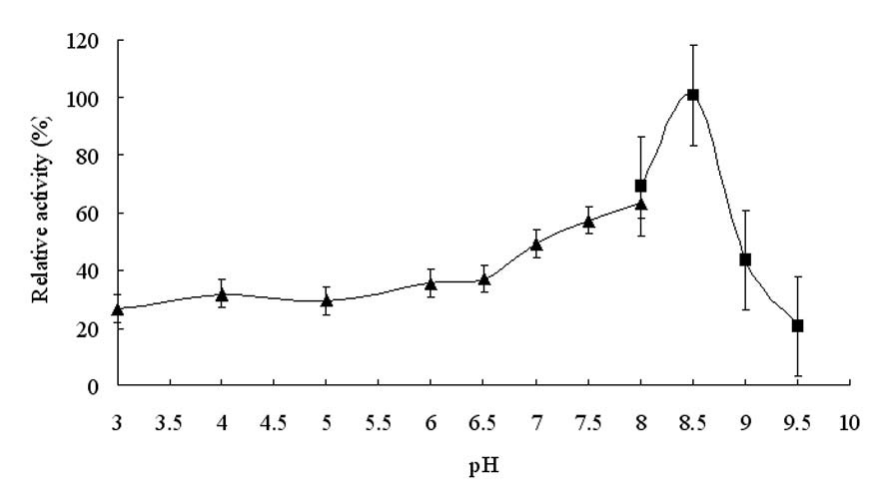
**Effects of pH on the enzymatic activity of recombinant FumF protein**. The buffers used were 50 mM citric acid/100 mM Na_2_HPO_4 _buffer (black regular triangle) (pH 3.0-8.0) and 100 mM glycine/NaOH buffer (black square) (pH 8.0-9.5). Relative activities represent enzyme activities at each pH divided by maximal activity.

**Figure 5:**
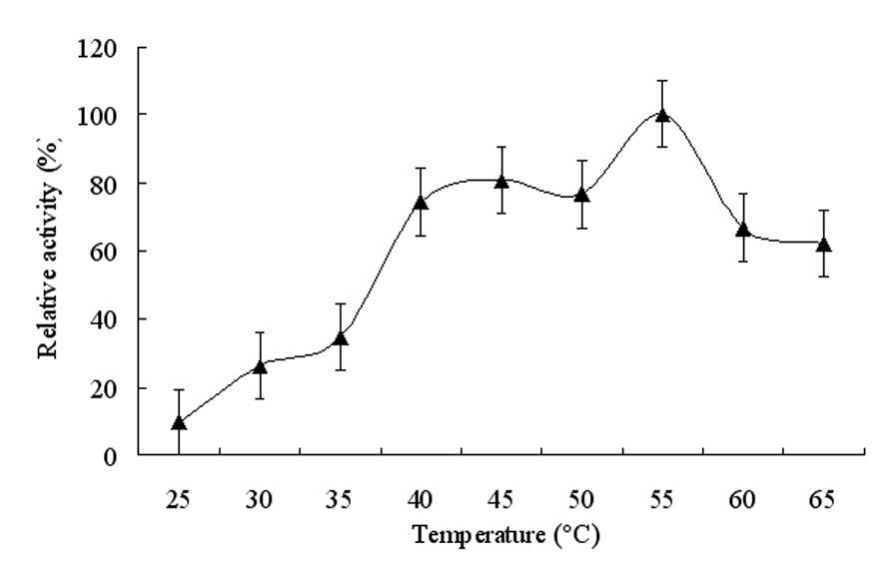
**Effects of temperature on the enzymatic activity of recombinant FumF protein**. Relative activities are the raw enzyme activities at each temperature divided by the maximal activity.

**Figure 6:**
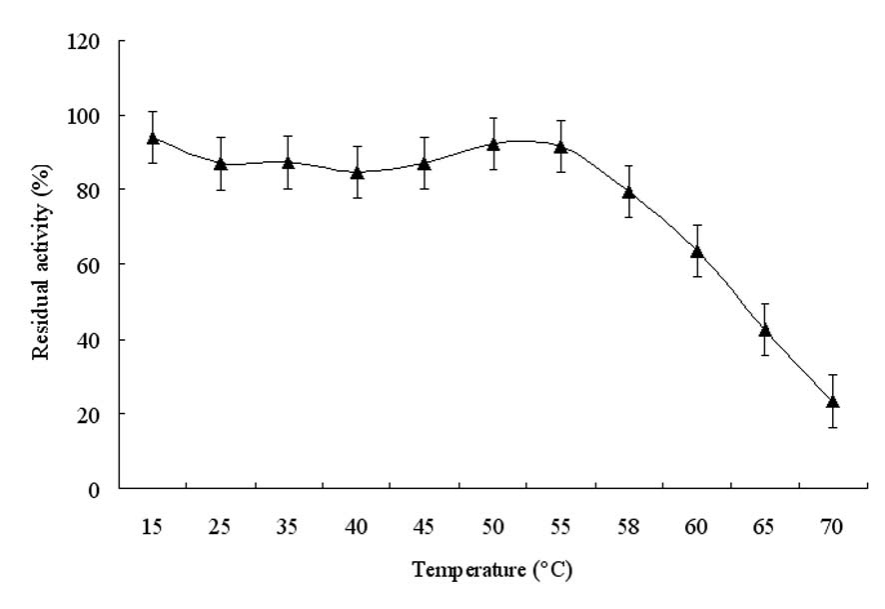
**The thermostability of recombinant FumF protein**. The enzyme was pre-incubated at temperature ranging from 15 to 70°C at optimum pH. Subsequently, the residual activity was determined with fumarate as the substrate at 55°C in 100 mM glycine/NaOH buffer (pH 8.5).

The enzymatic activity of the purified FumF protein was measured at various pHs from 6.5-9.5 to determine the optimal pH for fumarase activity. Activity of the recombinant enzyme was highest at pH 8.5 and dramatically suppressed below pH 7.0 and above pH 9.0 (Figure 4). The observed pH range of the recombinant enzyme was consistent with the reported properties of a thermostable Class II fumarase from *Thermus thermophilus *[21]. To determine the optimal temperature for the enzymatic reaction catalyzed by FumF, activity was measured at pH 8.5 over the temperature range 25-65°C. The enzyme demonstrated only 70% of its maximum activity below 40°C, but exhibited >70% of its maximum activity between 40-60°C, and maximal activity at approximately 55°C (Figure 5). This optimal temperature was about 5°C higher than that reported for stFumC protein from *Streptomyces thermovulgaris *[22]. To determine the thermostability of FumF, we incubated the purified putative fumarase in a water bath at temperatures between 15-70°C for up to 30 min and measured the residual activity with added substrate at pH 8.5 and 55°C. The activity of FumF was stable with increasing temperature below 58°C in the absence of any stabilizer (Figure 6). When the temperature was higher than 60°C, FumF protein lost almost 50% of its activity. This result suggests that FumF protein was derived from a thermophilic microorganism and is relatively stable below 60°C. Furthermore, the thermal properties of FumF protein suggest potential applications in biotechnology.

Class II fumarase activity is frequently influenced by divalent metal ions and non-ionic surfactants, so the effects of metal ions, EDTA, and SDS on the hydration activity of FumF were determined. Enzyme activity without added metal ions was taken as the baseline (100%). The presence of 5 mM MgCl_2 _stimulated enzyme activity to a maximum value of 110% of baseline, whereas NH_4_Cl, FeCl_2_, FeCl_3_, and ZnCl_2 _dramatically reduced the enzymatic activity to 77%, 63%, 52.6%, and 45%, respectively. By contrast, neither CaCl_2 _nor the calcium chelator EDTA (≤ 2 mM) altered enzymatic activity. When the concentration of EDTA was higher than 2.5 mM, enzyme activity was reduced by more than 20%. Enzyme activity was suppressed by 37% in CuCl_2_, while the anionic surfactant SDS reduced activity by 50% in the absence of metal ions. These results suggest that FumF is a Mg^2+^-dependent enzyme with optimal activity at 5 mM Mg^2+ ^and different with a fumarase from *Arabidopsis thaliana *[23]. The enzyme activity reached 726 U/mg protein using fumarate as the substrate under the optimal reaction conditions. The specific activity of the recombinant enzyme was higher than a fumarase from *Corynebacterium glutamicum *[2] and a stFumC protein from *Streptomyces thermovulgaris *[22]. These results also indicated that the active catalytic domain of FumF protein does not contain a potential metal ion binding site.

### Enzyme kinetics of recombinant FumF protein

The kinetic parameters of the recombinant FumF enzyme were determined using different fumarate concentrations. The initial rate of the enzyme reaction was measured under optimal reaction conditions. The reaction kinetic parameters of the purified enzyme were determined from double reciprocal Lineweaver-Burk plots. The putative fumarase had an apparent *K*_m _value of 0.48 mM, a *V*_max _value of 827 μM/min/mg, and a *k*_cat_/*K*_m _value of 1900/mM/s with fumarate as the substrate. Table 1 compares the properties of various fumarases from a number of different sources. FumF protein had a comparable *k*_cat_*/K*_m _value to most [7,24]. In comparison, the *K*_m _values of purified fumarases from other sources ranged from 0.25-1.0 mM (Table 1). The *K*_m _value for FumF protein was similar to that of the MmcBC fumarase from *Pelotomaculum thermopropionicum *strain SI (DSM 13744) [7]. A more complete analysis of the optimal reaction conditions and properties of FumF protein could enable better industrial production of L-malate under higher temperature conditions [25]. Furthermore, elucidating the catalytic mechanism through studies of the 3 D structures of FumF protein by X-ray diffraction could define protein regions that determine catalytic rates.

**Table 1:** Kinetic parameters of fumarases from various sources.

Substrate/Source	Fumarate			L-Malate		
	
	*K*_m _(mM)	*V*_max _(μmol product/min/mg enzyme)	*k*_cat_/*K*_m _(/mM/s)	*K*_m _(mM)	*V*_max _(μmol product/min/mg enzyme)	*k*_cat_/*K*_m _(/mM/s)
*Pelotomaculum thermopropionicum* (MmcBC) [7]	0.43	201	509	0.59	23.7	42.7
*Thermus thermophilus *AS15-1 (pH7.5) [21]	1.0	1340	NR	NR	NR	NR
*Bacterium *MPOB (DSM 10017) [24]	0.25	NR	690	2.38	NR	540
*Pyrococcus furiosus* (Pf fumarase) [25]	0.34	1376	3200	0.41	1892	3700
Marine samples (FumF, this work)	0.48	827	1900	3.15	282	100

NR, not reports.

## Conclusions

We constructed a plasmid library from marine water samples and cloned a novel fumarase gene (*fumF*) by a sequence-based screening strategy. Sequence analysis suggested that the identified gene product was related to prokaryotic Class II fumarases. A detailed biochemical characterization was also performed that encompassed the amino acid sequence, specific activity, pH-activity profile, and metal ion-activity profile. Identification of a new fumarase from marine microorganisms highlights the advantages of metagenomic libraries for cloning novel genes through sequence-based screening [26]. A similar approach can also be employed to find other biomolecules with applications in biotechnology.

## Methods

### DNA manipulation and protein analysis

All recombinant DNA techniques, including cloning and subcloning, transformation of *E. coli *cells, and PCR, were performed as described [27] or by following the manufacturer's instructions (unless otherwise indicated). Protein preparation and analysis, including protein extraction from *E. coli*, protein quantification, and SDS-PAGE, were performed as described in standard protocols [28].

### DNA extraction from marine sediment samples and construction of metagenomic library

Approximately 1000 L of surface seawater was collected from the South China Sea (21° 28' N, 109° 07' E) on 28 March 2007. The pH was 8.2, the temperature 15°C, and salinity 3.2%. Bacterial cells were collected by filtration on a membrane of 0.45 μm pore size (GE Whatman), recovered by washing the membrane with 1 × STE buffer, and concentrated by centrifugation at 12,000 rpm for 15 min. High molecular weight DNA from the sea samples was extracted based on a method described previously [29], with minor modifications. Briefly, the microbial DNA was washed twice with 75% (v/v) ethanol, air dried, and dissolved in 1 × TE buffer (10 mM Tris-HCl, pH 8.0 and 1 mM Na_2_EDTA). To remove contaminants, the DNA extract was further purified on a 0.6% (w/v) low-melting agarose gel run at 60 V for 3 h. The DNA fragments (≈ 23 kb) were recovered from the gel. The purified DNA was partially digested with *Eco*RI, followed by size fractionation with low-melting agarose gel as described [16]. Fractions containing DNA fragments of 1.0-15 kb were ligated into *Eco*RI-cleaved and dephosphorylated pGEM-3Zf (+) vector, and the ligation products were used to transfect competent *E. coli *DH5α. After overnight growth on LB agar plates containing 100 μg/ml ampicillin, white colonies harboring plasmids bearing inserts were collected and used to construct the metagenomic library. This library was stored at -80°C until screening.

### DNA sequence analysis, database search, and gene structure characterization

Sequence analysis was performed with the BigDye Terminator Cycle sequencing kit on an ABI Prism 3700 DNA analyzer (Applied Biosystems, USA). Protein translation was carried out with the Web-based translation tool at the Expasy homepage http://www.expasy.org/tools/dna.html. Sequence predictions were retrieved from the protein and nucleotide databases of the National Center for Biotechnology Information (NCBI) Entrez page http://www.ncbi.nlm.nih.gov/Entrez/. Sequence similarity searches were performed with BLAST 2.0. Amino acid sequence alignment of Undec1A with homologous proteins was conducted with Align X, a component of the Vector NTI suite (Informax, North Bethesda, MD, USA), using the blosum62mt2 scoring matrix. Based on comparison of the deduced amino acid sequence, a gene (*fumF*) encoding a putative novel fumarase was identified and further characterized. A phylogenetic tree was constructed using the neighbor-joining method with Molecular Evolutionary Genetics Analysis 3.1 (MEGA, Version 4.0) [30]. A boot-strapping value was used to estimate the reliability of phylogenetic reconstructions (1000 replicates).

### Expression and purification of the recombinant fumarase protein

The *fumF *nucleotide sequence was amplified from the plasmid (pGXAM3566) isolated from a clone with fumarase activity. Polymerase chain reaction (PCR) was carried out in a total volume of 50 μl containing 2.5 mM MgCl_2_, 10 mM Tris-HCl (pH 8.4), 0.2 mM of each dNTP, 0.4 μM of each primer, 1.0 unit Vent DNA polymerase (NEB, USA), and 10 ng plasmid template. Restriction enzyme sites (underlined) for *Bam*HI and *Hin*dIII were designed in the forward primer and reverse primers: (5'-TTATGGATCCGATGGATGTTGAAGCTG -3'/5'- GGCAAGCTTTAGGGCGAATTCCGG -3'). The PCR cycle consisted of an initial denaturation step of 96°C for 2 min, then 30 cycles of 94°C for 40 s, 57°C for 30 s, and 72°C for 2 min, followed by a final extension step at 72°C for 10 min. After amplification, the PCR product mixture was digested with *Bam*HI and *Hin*dIII, and ligated directly into pETBlue-2 (Novagen) expression vector cleaved with the same enzymes. The resultant recombinant plasmids were transferred into NovaBlue (Novagen) competent cells and plated onto LB selection plates. After overnight incubation at 37°C, positive white colonies were picked for isolation of the recombinant expression plasmid, which was subsequently introduced into *E. coli *BL21(DE3)pLysS (Novagen) to express the target protein.

The transformed bacterial cells were cultured in LB medium containing 50 μg/ml carbenicillin and 100 μg/ml chloramphenicol at 37°C, and protein expression was induced by the addition of 1 mM isopropyl-β-D-thiogalactopyranoside when the optical density at 660 nm reached 0.6. After incubation for an additional 6 h, the cells were harvested, washed twice with PBS (pH 7.6), and lysed by sonication in 10 ml of 20 mM Tris-HCl, pH 8.0. The lysate was centrifuged twice at 30,000 ×g for 20 min at 4°C, and 1 ml of the supernatant was diluted with 5 ml of column buffer (20 mM Tris-HCl, pH 8.0, 10 mM imidazole, 300 mM NaCl) and applied onto an equilibrated nickel nitrilotriacetic acid (Ni-NTA) column containing 1 ml of Ni-NTA-agarose (Novagen). The His-tagged FumF protein was expressed and purified using Ni-NTA agarose resin (Qiagen, Valencia, CA, USA), according to the manufacturer's instructions. The protein concentration was determined by a Bio-Rad protein assay kit using bovine serum albumin as the standard. The protein purified with Ni-NTA column and gel filtration was used for enzyme activity assays.

### Identification of the hydration product

The hydration product of the putative FumF was identified by high performance liquid chromatography (HPLC). Sodium fumarate (Sigma) was used as the substrate for characterization of the fumarase. The standard incubation mixture for fumarase activity consisted of 50 mM sodium phosphate buffer, pH 7.3, 10 mM sodium fumarate, and approximately 20 μl fumarase in 1 ml buffer. The final enzyme concentration was adjusted to approximately 1.5 μg/μl. The reaction was conducted in a total volume of 1.5 ml at 37°C for 15 min. Upon termination of the reaction, the residual protein was removed by centrifugation through a protein binding membrane (Vivaspin 500, Vivascience, Littleton, MA, USA). The filtered reaction sample was separated on a reverse phase C18 column (125 × 4 mm, Waters, USA) and UV detector at 210 nm [31]. The mobile phase was water of pH 2.10 to 2.15 (adjusted with perchloric acid) at a flow rate of 0.7 ml/min. The analysis was performed at 30°C.

### Partial physicochemical characterization of the recombinant fumarase protein

Unless otherwise specified, fumarase activity was determined spectrophotometrically by measuring the conversion of L-malate to fumarate or fumarate to L-malate at 250 nm and 37°C as described [32,33] with a few modifications. The reaction was initiated by the addition of 1.5 ml 50 mM L-malate or fumarate adjusted to pH 7.3 with NaOH. The increase in absorbance at 250 nm was measured as a function of time. One unit of enzyme activity was defined as the amount of the enzyme that converts 1 μmol substrate per min under standard assay conditions.

The profiles of activity versus pH and activity versus temperature were determined using the standard assay method. All experiments were performed in triplicate. To measure the effect of pH on the activity of the recombinant FumF protein, the enzyme activity was assayed over the pH range 3.0-8.0 in 50 mM citric acid/100 mM Na_2_HPO_4 _buffer, while over pH 8.0-9.5, the activity was assayed in 100 mM glycine/NaOH buffer. To assess the effect of temperature on FumF activity, the enzyme was assayed at various temperatures (25 to 60°C) in 100 mM glycine/NaOH buffer (pH 8.5). Thermostability was determined by pre-incubating the purified enzyme for up to 30 min at temperatures ranging from 15 to 70°C in 100 mM glycine/NaOH buffer (pH 8.5) and analyzing the residual activity with fumarate as the substrate [34,35]. Various metal compounds (NH_4_Cl, MgCl_2_, CuCl_2_, CaCl_2_, MnCl_2_, ZnCl_2_, FeCl_2_, FeCl_3_, AlCl_3_), a chelating agent (ethylenediamine tetraacetic acid, EDTA), and surfactant (sodium dodecyl sulfate, SDS) were added to optimal reaction systems to investigate their effects on enzyme activity. Unless otherwise specified, the concentrations were 0.5-10 mM metal ions, 2 mM EDTA, and 1% (v/v) SDS.

### Enzyme kinetic assays

Enzyme kinetic parameters of FumF protein were determined by measuring the rate of fumarase hydration at various concentrations (0.1-5.0 mM) and dehydration of L-malate at various concentrations (0.07-5.5 mM) at 55°C in 100 mM glycine/NaOH buffer (pH 8.5). The final enzyme concentration was adjusted to approximately 15 μM. The enzyme kinetic parameters *K*_m _and *V*_max _were determined from Lineweaver-Burk plots using the Enzyme Kinetics computer program [36].

### Nucleotide sequence accession number

The *fumF *nucleotide sequence has been deposited in the GenBank under accession number GQ353347.

## List of abbreviations used

FumA: fumarase A; FumB: fumarase B; FumC: fumarase C; SDS-PAGE: sodium dodecyl sulfate-polyacrylamide gel electrophoresis; *E. coli*: *Escherichia coli*; Ni-NTA: nickel-nitrilotriacetic acid; HPLC: high performance liquid chromatography; EDTA: ethylenediaminetetraacetic acid; SDS: sodium dodecyl sulfate; ORF: open reading frame.

## Competing interests

The authors declare that they have no competing interests.

## Authors' contributions

CJJ, BW and XLT have set up and designed the study; CJJ, LLW and GCZ have set up and performed all experiments; KJ and SXL have performed the experiments of construction library; ZYH and GFM have performed cloning, expression and purification experiments; FFL and PHS have performed bioinformatic analysis; GQH has performed the HPLC detection experiments; LLW and GCZ have set up and performed the biochemical characterization experiments; CJJ and XLT have analyzed the data and written the manuscript; WLK, XMQ and YLB have rechecked all the enzymatic data. All authors discussed the results and commented the manuscript and all authors read and approved the final manuscript.

## Supplementary Material

Additional file 1 **Construction metagenomic library of marine uncultivated microorganisms from the South China Sea**. Lane 1: 1kb ladder marker; lane2-12: *Eco*RI-digested plasmids of the random clones from the metagenomic library. Click here for file

Additional file 2 **HPLC chromatograph of the hydration of fumarate to form L-malate catalyzed by the recombinant FumF protein**. The data indicated the formation of L-malate from fumarate in 50 mM sodium phosphate buffer (pH 7.3) with the recombinant FumF protein. Click here for file
