# Supplementary figures and images for: Identification and characterization of a novel fumarase gene by metagenome expression cloning from marine microorganisms
Source: Microb Cell Fact. 2010 Nov 23;9:91. doi: 10.1186/1475-2859-9-91 (PMC3002918; doi:10.1186/1475-2859-9-91)

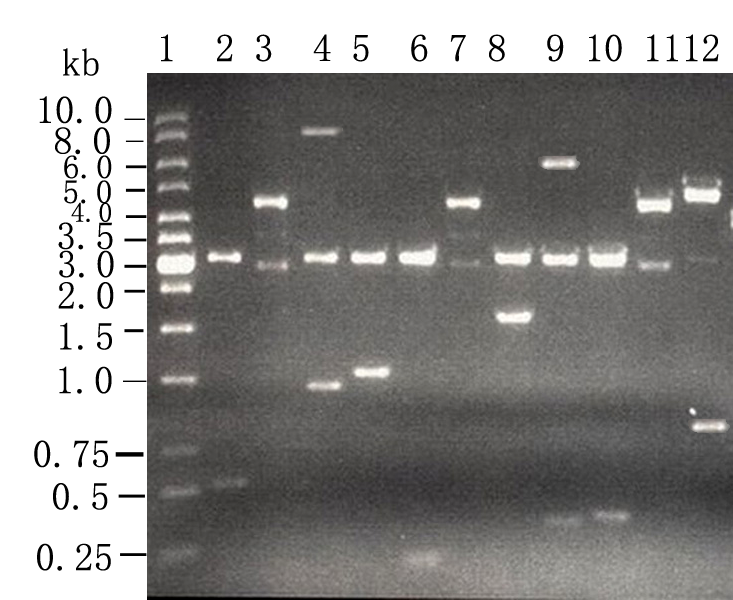

Supplement: Additional file 1 — Construction metagenomic library of marine uncultivated microorganisms from the South China Sea. Lane 1: 1kb ladder marker; lane2-12: EcoRI-digested plasmids of the random clones from the metagenomic library. [file 1475-2859-9-91-S1.TIFF]

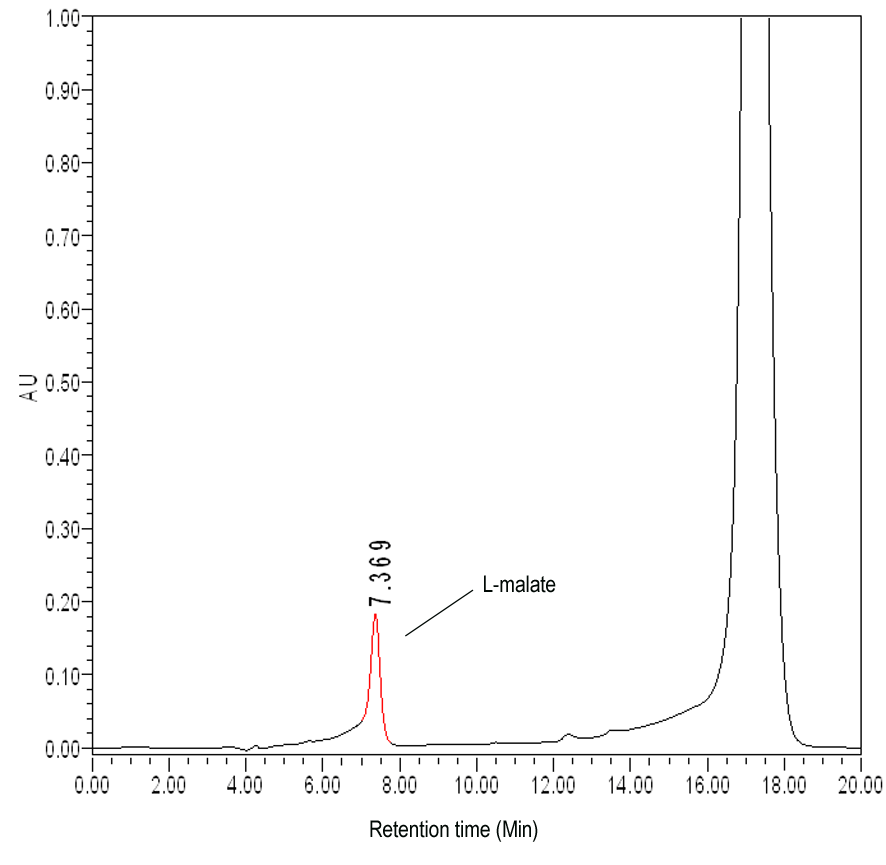

Supplement: Additional file 2 — HPLC chromatograph of the hydration of fumarate to form L-malate catalyzed by the recombinant FumF protein. The data indicated the formation of L-malate from fumarate in 50 mM sodium phosphate buffer (pH 7.3) with the recombinant FumF protein. [file 1475-2859-9-91-S2.TIFF]
